# Understanding molt control switches: Transcriptomic and expression analysis of the genes involved in ecdysteroidogenesis and cholesterol uptake pathways in the Y-organ of the blue crab, *Callinectes sapidus*

**DOI:** 10.1371/journal.pone.0256735

**Published:** 2021-09-03

**Authors:** Elena Legrand, Tsvetan Bachvaroff, Tracey B. Schock, J. Sook Chung

**Affiliations:** 1 Institute of Marine and Environmental Technology, University of Maryland Center for Environmental Science, Baltimore, Maryland, United States of America; 2 Chemical Sciences Division, Hollings Marine Laboratory, National Institute of Standards and Technology, Charleston, South Carolina, United States of America; Stazione Zoologica Anton Dohrn, ITALY

## Abstract

The crustacean molting process is regulated by an interplay of hormones produced by the eyestalk ganglia and Y-organs (YO). Molt-inhibiting hormone and crustacean hyperglycemic hormone released by the sinus gland of the eyestalk ganglia (EG) inhibit the synthesis and secretion of ecdysteroid by the YO, hence regulating hemolymph levels during the molt cycle. The purpose of this study is to investigate the ecdysteroidogenesis pathway, specifically genes linked to changes in ecdysteroid levels occurring at early premolt (ePM). To this end, a reference transcriptome based on YO, EG, and hepatopancreas was *de novo* assembled. Two genes (cholesterol 7-desaturase *Neverland* and cytochrome p450 307a1-like *Spook*) involved in ecdysteroidogenesis were identified from the YO transcriptome using sequence comparisons and transcript abundance. Two other candidates, *Hormone receptor 4* and *probable cytochrome p450 49a1* potentially involved in ecdysteroidogenesis were also identified. Since cholesterol is the ecdysteroid precursor, a putative cholesterol carrier (*Apolipoprotein D-like*) was also examined to understand if cholesterol uptake coincided with the increase in the ecdysteroid levels at the ePM stage. The expression level changes of the five candidate genes in the YO were compared between intermolt (IM) and induced ePM (iePM) stages using transcriptomic analysis. Expression analysis using qPCR were carried out at IM, iePM, and normal ePM. The increase in *Spook* and *Neverland* expression in the YO at the ePM was accompanied by a concomitant rise in ecdysteroid levels. The data obtained from iePM stage were congruent with those obtained from the normal ePM stage of intact control animals. The present findings support the role of Halloween genes in the ecdysteroidogenesis and molt cycle in the blue crab, *Callinectes sapidus*.

## Introduction

Crustacean molting, required for somatic growth, is regulated by hormones produced by the eyestalk ganglia (EG) and Y-organs (YO). The ganglia, located within the eyestalk of decapod crustaceans, contains the primary endocrine tissue, the X-organ medulla terminalis-sinus gland complex. This tissue produces and releases the four crustacean hyperglycemic hormone family members: molt-inhibiting hormone (MIH), crustacean hyperglycemic hormone (CHH), mandibular-organ inhibiting hormone, and gonad/vitellogenesis-inhibiting hormone [1–3]. Together with a multifunctional CHH, MIH suppresses YO activity, keeping ecdysteroid (Ecd) in the hemolymph low at the intermolt (IM) stage [3–7]. The injection of MIH and CHH into eyestalk-ablated animals can substitute for eyestalk tissue itself, suppressing hemolymph Ecd [4,5,7]. Hemolymph Ecd levels in turn influence MIH transcription in EG of the blue crab, *Callinectes sapidus* [8,9], possibly through tissue-specific isoforms of ecdysone/retinoid X receptors [10]. An interaction between the two tissues for molt control warrants further study on what changes occur in the YO from IM to premolt (PM), specifically leading to increased hemolymph Ecd.

With cholesterol as the precursor molecule, the YO produces two inactive forms of Ecd: α-ecdysone and 25-deoxyecdysone (25dE), which are converted in the peripheral tissues into the active forms of 20-hydroxyecdysone (20E) and 25-deoxy-20 hydroxyecdysone or Ponasterone A (PonA). The hydroxylation reactions are catalyzed by cytochrome P450 mono-oxygenases encoded by *shade*. The conversion process is partial, indicated by the measurable levels of PonA, 3-dehydro-20-hydroxyecdysone (3d20E), 20-hydroxyecdysone (20E) and 25- deoxyecdysone (25dE) in the hemolymph [4]. In *C*. *sapidus*, PonA is the principal Ecd in the hemolymph of PM animals, followed by 20E. As alluded to earlier, eyestalk neuropeptide MIH and/or CHH in Ecd synthesis is a key regulator because eyestalk-ablated animals at the PM stage carry 20E as the major Ecd in the hemolymph, not PonA [4].

Hemolymph Ecd concentrations being cyclic repeatedly rise and fall during the molt cycle of several crustacean species including the blue crab, *C*. *sapidus* [4], the American lobster, *Homarus americanus* [11], and the tanner crab, *Chionoecetes bairdi* [12]. Although the hemolymph Ecd concentrations differ by species, the described pattern is that animals at the IM stage have low levels of Ecd ranging from 0 ng/mL-100 ng/mL, while those at PM have much higher levels with 200 ng/mL to 2500 ng/mL [11]. What exactly triggers the changes in Ecd concentrations remains to be unraveled in decapod crustaceans.

Since Ecd synthesis starts with cholesterol as the precursor, cyclic fluctuation of Ecd concentrations during the molt cycle implies changes in cholesterol levels [13–15]. Hence, cholesterol uptake and transport are considered the critical, rate-limiting step for Ecd synthesis in arthropods, as they do not synthesize cholesterol *de novo* [16]. In vertebrates, cholesterol uptake and transport are facilitated by the lipoproteins such as Low-Density-Lipoprotein or apolipoprotein (Apo), through a receptor-mediated endocytic pathway, Low-Density-Lipoprotein-receptor [17]. Insect lipoprotein is involved in cholesterol uptake by the insect prothoracic gland [18], suggesting that cholesterol-binding protein and its receptor expression may be positively associated with cholesterol transport and Ecd synthesis during the molt cycle. A hormone that is an equivalent of insect prothoracic gland hormone has not yet been found in crustaceans.

The Ecd synthesis pathway in insects involves the Halloween gene family, encoding cytochrome P450 enzymes [19,20]. The Halloween gene family catalyzes several ecdysone synthesis steps described in *Drosophila melanogaster* [21–24]. Most Halloween genes are found among other arthropod species. For example, the water flea, *Daphnia pulex* has orthologs of all 5 of these genes [25] while four (cytochrome P450 (*CYP*), *307A1* (*spook)*, *CYP315A* (*shadow)*, and *CYP306A1* (*phantom*)) are found in the swimming crab, *Portunus terituberculatus* [26] and the kuruma prawn, *Marsupenaeus japonicus* [6]. However, while *spook* and *spookier* have been detected in arthropods, potentially involved in the second step of the Ecd synthesis (transformation of 7-dehydrocholesterol (7dC) to Δ4-Diketol and Diketol), only a *spook* homolog has been identified in crustaceans [27].

In this study, we aimed to determine the increase in the YO activity, especially changes in the expression level of genes involved in crustacean Ecd synthesis during the transition from IM to PM, specifically when the *C*. *sapidus* physiologically commit to the next molt cycle. To this end, we first compared the transcriptomic data of the YO of intact animals with those of eyestalk-ablated ones (induced early premolt iePM stage) and identified the genes that are potentially involved in early ecdysteroidogenesis and cholesterol transport/uptake. We then measured the distribution of these genes across all crab tissues. And finally, we measured the gene expression in the YO of animals at different molt stages and in eyestalk-ablated animals, together correlating with the Ecd and cholesterol concentrations in the hemolymph.

## Materials and methods

### Animal culture

The blue crab, *C*. *sapidus*, was produced in the Blue Crab Hatchery, Aquaculture Research Center (ARC, Institute of Marine and Environmental Technology, Baltimore, MD, USA). The animals were reared in individual compartments (15 cm × 15 cm) in a closed recirculating aquaculture system with 25 ppt artificial seawater at 22–23°C [8]. They were fed daily with a piece of squid (10% to 20% of body weight) between 4–6 P.M. EST until they reached the target experimental size (>70 mm). All animals were sacrificed in the afternoon (2–6 P.M.). Water quality was examined daily by the ZooQuatic Laboratory LLC (Baltimore, MD).

### Eyestalk ablation experiment

Adult *C*. *sapidus* females and males at IM (n = 5–7) were used for bilateral eyestalk ablation. In brief, hemolymph (50 μL) was directly withdrawn into a 28.5 gauge insulin syringe containing 50 μL of a modified crustacean anticoagulant [28] (pH 7.4) at a 1:1 ratio (volume fraction), for basal Ecd levels at t = 0 [29]. The animals were chilled by placing them on ice for 5 min, and both eyestalks were then removed. The bleeding through the cut was monitored, ensuring the survival of the animal. The ablated animals were returned to their compartment and maintained for 7 days, with hemolymph sampling on days 3 and 7. After sampling at t = 7 days post-ablation, the animals were sacrificed for tissue dissection. The YOs were dissected in ice-cold diethylpyrocarbonate-treated crustacean saline [30] under a stereomicroscope (Leica), frozen immediately on dry ice and stored at -80°C until further analysis. EG and hepatopancreas were also collected in male *C*. *sapidus* following the same procedure.

### Prepuberty molt cycle

Juvenile females at the prepuberty stage (undergoing pubertal terminal molt, [31]) were sampled (hemolymph and YO) at IM (n = 8), ePM (n = 15), and PM (n = 11) stages as described above, for RT-PCR analysis.

### RNA extraction, library preparation and Illumina sequencing

Total RNAs from the YO, EG, and hepatopancreas of male *C*. *sapidus* at 7 days post-ablation (iePM) and YO from female *C*. *sapidus* were extracted using Qiazol following the manufacturer’s recommendations. RNA concentrations were measured using a Nanodrop. RNA samples from 3 to 5 individuals were pooled and submitted to Macrogen (www.macrogenusa.com) for RNA sequencing, where the quantity and quality of RNA for each sample was checked using a Bioanalyzer (Agilent).

### *De novo* assembly and quantification

Four RNAseq read sets were generated: EG, hepatopancreas, and the YO at IM and iePM. The quality of the raw reads was evaluated with FastQC. The raw reads were pre-processed using Trimmomatic to remove reads containing adapter, reads containing poly-N, and low-quality reads [32]. Clean reads were assembled as a reference transcriptome using Trinity (v2.6.6) with default parameters [33]. The N50 parameter and the % mapped reads to the assembly using bowtie2 were assessed. A clustered transcriptome was built using CD-HIT-EST v4.6.6 with 0.95% identity [34]. The completeness of the clustered and unclustered assemblies were evaluated using Benchmarking Universal Single-Copy Orthologs (BUSCO) analysis [35]. BUSCO v3.0 was used with the dependencies augustus-3.2.1 and hmmer-3.2.1 to compare the transcriptomes against the arthoropoda_odb9 lineage dataset with an e-value threshold of 1e-05. Trimmed reads obtained from each tissue were mapped back to the reference transcriptome using Bowtie2 v2.1.0. The abundance of transcripts and genes were evaluated using RSEM v1.2.28 and calculated as Transcripts Per Million (TPM) values [36]. A transcript was considered expressed using the threshold of TPM ≥ 1. Unique and shared transcripts were identified using an UpSet plot [37] generated by the web-based tool (https://gehlenborglab.shinyapps.io/upsetr/).

### Functional annotation and candidate genes

Transcript abundance was used to explore the YO transcriptomes (IM and iePM). The most highly expressed transcripts of the YO at iePM (TPM ≥ 100) were aligned against the subset taxonomy group "Arthropods" of the NCBI non-redundant (nr) protein database of using blastx-fast with an e-value cut off of 1.0e-5 using Blast2go suite in OmicsBox [38]. Contigs were functionally annotated following Blast2go workflow using Gene Ontology and completed by an eggnog annotation in OmicsBox [39]. The genes of interest were further curated manually using BLAST. The genes involved in the ecdysteroid synthesis and cholesterol uptake and transport that were found based on GO terms and sequence descriptions were selected for further analysis.

### Sequence homology and phylogenetic tree analysis

The mRNA sequences were translated using the ExPasy (https://web.expasy.org). The predicted open reading frames were aligned against invertebrate sequences for homology and phylogeny. Phylogenetic tree was generated using Phylogeny.fr [40]. Sequences were aligned using MUSCLE, GBLOCKS site selection, and presented fasta format as supplemental data (S1–S5 Files). For each alignment, the sequences from *Callinectes sapidus*, were compares using blastp to find the best hit to: *P*. *trituberculatus*, *S*. *paramamosain*, *H*. *americanus*, *P*.*monodon*, *P*. *vannamei*, *Hyalella azteca*, *Daphnia magna*, *Apis mellifera*, and *Drosophila melanogaster*, followed by alignment and phylogenetic analysis as described above. The annotations were confirmed by comparisons with the canonical genes described in *D*. *melanogaster*. In one case, for the Apolipoprotein D-like gene a single, canonical sequence from *D*. *melanogaster* was not available, so metazoan outgroups were used.

### Tissue distribution in an adult female *C*. *sapidus*: Primers and sequence verification

The expression and sequence of these genes were confirmed using endpoint PCR after candidates were identified in the YO transcriptome. A pair of primers for each gene (Table 1) were generated using NCBI Primer-BLAST based on the transcriptomic sequences. PCR templates were cDNA containing 12.5 ng total RNA equivalent from 17 different tissues in a single adult female *C*. *sapidus* at IM and ovarian stage 2. The PCR conditions were as follows: initial denaturation 94°C for 2.5 min followed by 35 cycles at 94°C for 30 s; 58°C for 30 s and 72°C for 1.5 min. The PCR products were analyzed on 1.5% agarose gel and stained with ethidium bromide. The PCR amplicons with the expected size were cloned and sequenced for confirmation as described [41,42].

**Table 1:** List of primers that were used for the tissue distribution in *C*. *sapidus*.

Gene name	Name	Sequence (5’-3’)
*Spook*	Spo-F1	TCCTTCAGGAACCTCTCTGGCATGA
	Spo-R1	CCTCAACGACGACAAAGAGCAGTGA
*Neverland*	Nvd-F1	GTGGCTATTGCGTGTCGTGTGATGT
	Nvd-R2	CTCTGAGGTGCTTGTGGTGGTGAAG
*Hormone receptor 4*	HR4-F1	GCGGGCGTGAACGACATCAAAAG
	HR4-R2	CTTCTTCATGGACAGCGGGCGAG
*cyp49a1*	cy49-F1	TAACACCACGAGCTTGGACAGCG
	cy49-R2	GATAGATGCGACCGCCACCACTC
*Apolipoprotein D-like*	AD-F1	CATTCATGCCAAGACCTCCAGCTCC
	AD-R2	GAGTAGCGGGCAGCGTCTATGTTT

### RNA extraction, cDNA synthesis and quantitative RT-PCR assay

Total RNAs of YO from the prepuberty females at different molt stages (IM n = 8, ePM n = 6, PM n = 8) were extracted as described above and were quantified with a Nanodrop Lite Spectrophotometer (Thermo Scientific). Reverse transcription with 1 μg to 1.5 μg total RNA was carried out using a Takara-PrimeScript RT reagent Kit with gDNA Eraser (Clontech). After diluted to a final concentration of 12.5 ng/μL, the quality of each cDNA sample was examined by amplifying the *AK* gene [8,10]. The standards for the genes were generated as described [43–45]. Briefly, a plasmid DNA containing each gene PCR product (generated using primers in Table 1) was generated and diluted to range from 200 to 2 million copies/well. The average amplification efficiency of the standards and samples were -3.521 ± 0.152 (n = 12) and R^2^ value of 0.995 ± 0.005 (n = 12, S1 Table). For expression levels of each gene (the primers listed in Table 2), the cDNA samples were assayed in duplicate using Fast SYBR Green Master Mix (Applied Biosystems) on an Applied Biosystems 7500 Real-Time PCR instrument. The melting curve analysis was included at the end of each assay, with expected melting temperature (Tm) ranged from 78 (*ApoD*), 80 (*Spook* and *cyp49a1*) to 81 (*Neverland* and *HR4*).

**Table 2:** List of primers that were used for the qPCR assays.

Gene name	Name	Sequence (5’-3’)
*Spook*	Spo-F3	CCACTTCTCCTCCTCGTTGGTTGTC
	Spo-R3	CCTGCAGCCTCTCTTCACCTCATAC
*Neverland*	Nvd-F2	CCAGTCCCCAATGTGCAGAGAGAAG
	Nvd-R2	CTCTGAGGTGCTTGTGGTGGTGAAG
*Hormone receptor 4*	HR4-F3	CGTCGGGAAGGAACGTCTTGTGT
	HR4-R2	CTTCTTCATGGACAGCGGGCGAG
*Cyp49a1*	cy49-F3	AACGTCTGAGGGCGTGGACAATG
	cy49-R3	CTATTTTGTGCCTGCTGGGGCCT
*Apolipoprotein D-like*	AD-F1	CATTCATGCCAAGACCTCCAGCTCC
	AD-R1	GAACGGGGCAAAGAAGCGTTCAATC

### Ecdysteroid Radioimmunoassay (Ecd-RIA)

An Ecd-RIA assay with an ecdysone-specific antiserum and ☯^3^H] PonA (Perkin Elmer) was used to measure the concentrations of total hemolymph Ecd in each stage of intact (IM, n = 8; ePM, n = 7, and PM, n = 11) and ablated animals (IM, n = 5 and iePM, n = 5) as described in [4]. Hemolymph (10 μL, 1:1 ratio of hemolymph: anticoagulant [28]) samples were assayed in duplicate. The results were analyzed with the AssayZap program (Biosoft).

### Cholesterol assay

Free cholesterol and cholesteryl ester were measured using a fluorometric Amplex Red Cholesterol Assay Kit by following the manufacturer’s instructions (Invitrogen). Briefly, 10 μL of hemolymph (a 1:1 ratio of hemolymph: anticoagulant [28]) from each animal at the following molt stages (IM, n = 8; ePM n = 15; PM, n = 11; IM n = 4; and iePM, n = 5) was diluted in 40 μL of reaction mix previously prepared, with the cholesterol standard ranging from 1 μg/mL to 8 μg/mL. The Amplex Red reagent was added to the wells, and the plates were incubated in the dark for 30 min at 37°C. Fluorescence was measured at 560 nm with an excitation at 590 nm immediately after incubation (SpectraMax M5). All samples, blanks, and standards were measured in duplicate.

### Statistical analysis

Statistical analyses were performed using the R software package (v3.4.3). When two groups were compared, Student’s *t*-test was calculated and significance was noted as follows:”*” *P*<0.05 and “**” *P*<0.01. For multiple group comparisons, a non-parametric Kruskal-Wallis test followed by Dunn post-hoc test with Holm adjustment was performed. The significance at *P*<0.05 was noted as”*” or letters.

## Results

### Sequencing and read assembly

**Fig 1:**
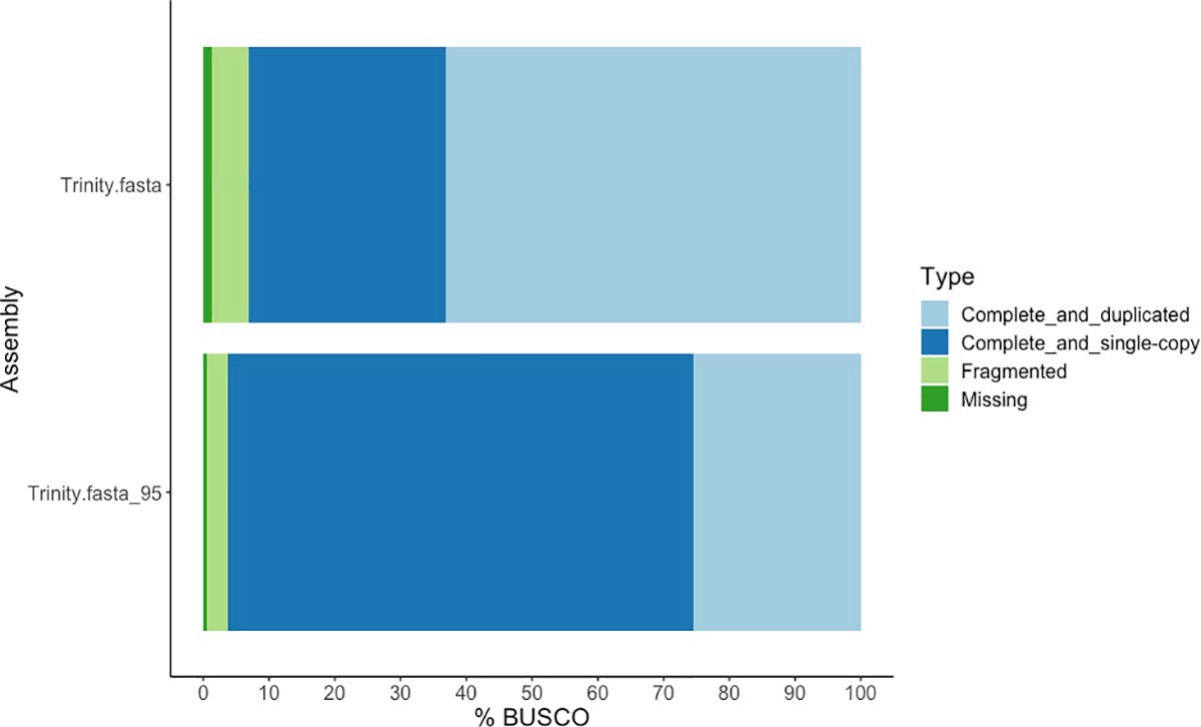
BUSCO assessment results of the Trinity and Trinity_95 transcriptome against the Arthropoda reference.

A reference transcriptome was *de novo* assembled using Trinity, including the YO transcriptome at IM and iePM stages, eyestalk ganglia and hepatopancreas (total 161M reads). A total of 377,335,498 bases were assembled in 260,172 Trinity genes and 362,308 Trinity transcripts (Table 3). BUSCO analysis of the transcriptome against the arthropod database of 1,066 genes (Fig 1) revealed 93% complete with 1% missing. Among the complete genes, the majority (68%) were duplicated. The redundancy of the Trinity assembly was then filtered using CD-HIT-EST, resulting in 260,148 Trinity genes (99% retained) and 313,761 transcripts (87% retained) (Table 3). The BUSCO analysis of the resulting Trinity_95 transcriptome improved the completeness to 96%, decreased duplicates from 67.8% to 26.5%, increased single-copy genes from 32.2% to 73.5% and reduced missing BUSCO to 0.5% (Fig 1). The Trinity_95 assembly was then used as the reference transcriptome for further analysis.

**Table 3:** Summary of Illumina sequencing statistics and mapping.

***Trinity assembly statistics***	
Total read bases (bp)	377,335,498
Number Trinity genes	260,172
Number Trinity transcripts	362,308
Average contig (bp)	1041.48
GC (%)	45.23
N50	2,854
***Trinity_95 assembly statistics***	
Total read bases (bp)	230,936,077
Number Trinity genes	260,148
Number Trinity transcripts	313,761
Average contig (bp)	736.03
GC (%)	44.37
N50	1,510
***YO transcriptome IM***	
Total number reads	33,758,864
% reads mapped to Trinity_95	83.66%
Total number of contigs	106,152
Number of filtered contigs (TPM> = 1)	40,605
Average filtered contig (bp)	1322.182
***YO transcriptome iePM***	
Total read bases (bp)	52,953,709
% reads mapped to Trinity_95	81.64%
Total number of contigs	119,502
Number of filtered contigs (TPM> = 1)	46,240
Average filtered contig (bp)	1266.056

**Fig 2:**
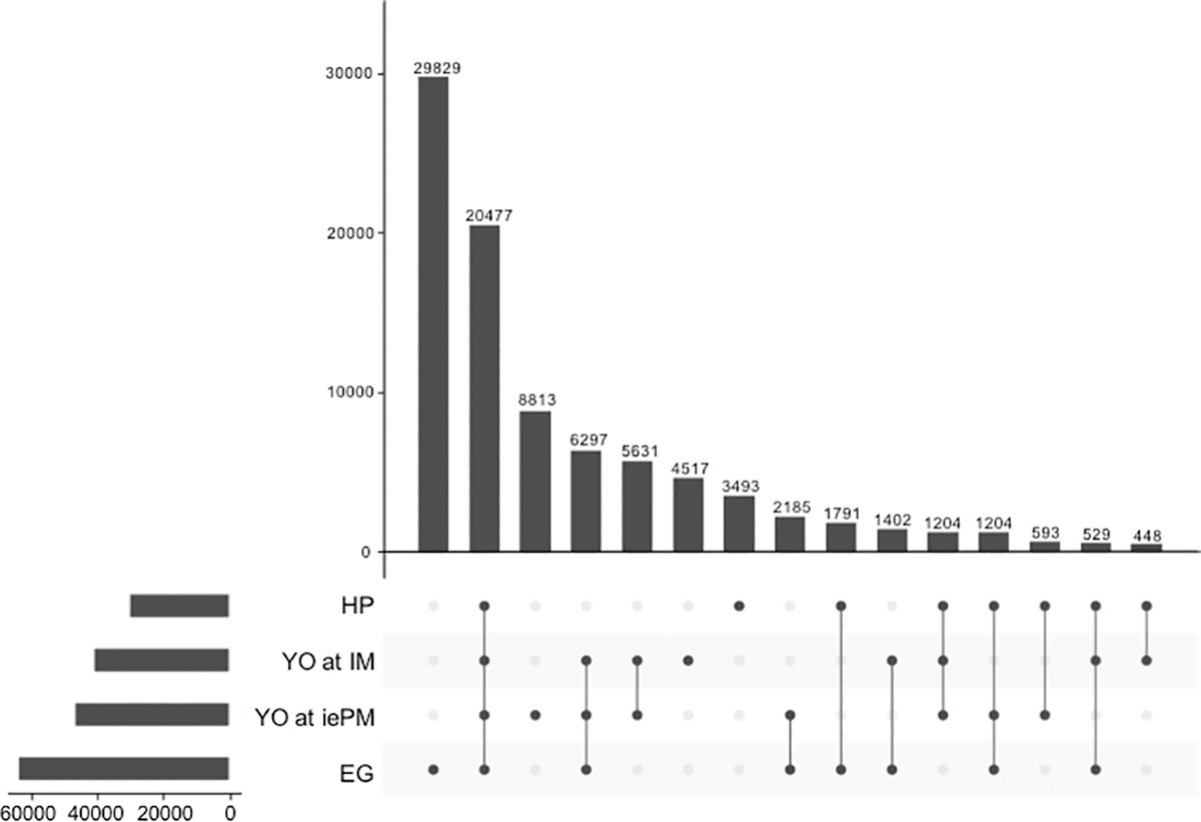
UpSet plot depicting the number of unique and shared transcripts with TPM ≥ 1 in each tissue. Intersection size represents the number of transcripts for each designated sets or groups. The dots and lines illustrate the groups defined as: HP: Hepatopancreas, YO at IM: Y-organ at intermolt, YO at iePM: Y-organ at induced early premolt and EG: Eyestalk ganglia.

Individual read sets of each tissue were mapped back to the Trinity_95 reference transcriptome: 33,758,864 and 52,953,709 reads for the YO at IM and iePM, respectively (Table 3). For the YO, 83.66% (IM) and 81.64% (iePM) of the reads were successfully aligned. Based on a transcript per million (TPM) cut off value ≥1 using RSEM, the EG transcriptome had the most transcripts with 63,714 contigs followed by the YO at iePM with 46,240 transcripts, 40,605 sequences in the YO at IM (15% and 13% of total assembly respectively) and 29,739 transcripts in the HP (Fig 2). The UpSet plot showed the intersection between the four transcriptomes. The four tissues had 20,477 sequences in common. In total, the YO at IM and iePM shared 33,609 sequences. EG has the most unique transcripts with 29,829 sequences followed by YO at iePM with 8,813 transcripts, YO at IM with 4,617 contigs and 3,493 transcripts in hepatopancreas. The YO data sets have been submitted o NCBI Sequence Read Archive (SRA) under the BioProject ID PRJNA701676.

### Functional annotation and genes involved in the molting process

The data from the YO transcriptomes were presented in the context of the molting process. The transcriptome at the iePM stage was investigated by focusing on the 608 highly expressed sequences with TPM>100 (max TPM of 269,633.82 for IM and 174,208.76 for iePM). Contigs involved in the ecdysteroidogenesis, together with cholesterol transport and uptake pathways, were searched using the following GO terms: "Process: ecdysteroid biosynthetic process," " ecdysone biosynthetic process", "Function: cholesterol binding," and "Process: regulation of cholesterol transport."

Among the ten genes retrieved (Table 4), four belonged to the Halloween genes: *Neverland (*cholesterol 7-desaturase; accession No MW556747*)*, *Spook (*cyp307a1-like; accession No MW556746*)*, *Disembodied (*cyp302a1), and *Shadow* (cyp315a1*)* based on reciprocal best hits when using *Drosophila* and the blue crab transcriptome.

**Table 4:** Abundance of genes in transcript per million (TPM) and their corresponding fragments per kilo base per million mapped reads (FPKM) involved in ecdysteroid synthesis, cholesterol uptake and transport in the YO transcriptomes of C. sapidus at intermolt (IM) and induced early premolt (iePM). When a gene presented several transcripts, the abundance of both transcripts is shown.

	TPM		FPKM	
Gene name	IM	iePM	IM	iePM
*Spook*	7143.87	15715.33	6787.59	14527.49
*Neverland*	526.73	1078.48	500.46	996.96
*Disembodied*	620.65	1239.28	589.70	1145.61
*Shadow*	261.74	194.21	248.68	179.53
	173.5	216.2	164.85	199.86
*Apolipoprotein D-like*	34.8	129.79	33.06	119.98
*D-β-hydroxybutyrate dehydrogenase*	139.51	237.98	132.55	219.99
*NPC1*	23.02	106.18	21.87	98.16
	47.44	175.65	45.08	162.38
*Hormone receptor 4*	114.73	222.27	109.01	205.47
*Adrenodoxin*, *mitochondrial*	31.17	166.65	29.61	154.05
*Probable cytochrome P450 49a1*	345.75	933.15	328.51	862.62
	573.37	847.27	544.77	783.23

At the iePM, *Spook* was the most highly expressed Halloween gene as it was the 3rd most abundant transcript in the iePM transcriptome. Three of four Halloween genes (*Spook*, *Neverland*, *and Disembodied)* showed two-fold increase in the YO from IM to iePM (Table 4). *Shadow* presented two isoforms with different expression patterns. Three genes involved in cholesterol transport and uptake (*Apolipoprotein D-like*, accession No MW556748; *D-β-hydroxybutyrate dehydrogenase*, and *NPC1*) were identified among the most expressed genes in the transcriptome (Table 4). *NPC1* presented the highest increase at iePM with a mean fold change of 4.2 followed by *Apolipoprotein D-like* and *D-β-hydroxybutyrate dehydrogenase*, a fold change of 3.7 and 1.7, respectively. Genes involved in ecdysteroidogenesis or cholesterol transport and uptake (*Hormone receptor 4*, accession No MW556749 and *Adrenodoxin*, *mitochondrial)* also increased from IM to iePM (1.9 and 5.3, respectively). Additionally, another cytochrome P450: *probable cytochrome P450 49a1* (accession No MW556750) was also identified as highly expressed in the transcriptome with a mean fold change of 2.1.

### Phylogenetic tree and sequence homology of candidate genes

Five of the ten genes described above were chosen for further investigation in *C*. *sapidus* YO based on their function and expression in the YO transcriptomes. These genes were involved in the two first steps of ecdysteroidogenesis (*Neverland* and *Spook)*, an additional cytochrome p450 (*probable cytochrome p450 49a1*), one gene involved in the cholesterol transport (*Apolipoprotein D-like)*, and *Hormone receptor 4*.

*Neverland’s* Rieske iron-sulfur domains (cd03531, pfam00355) were identified in the *C*. *sapidus’* sequence (Table 5). Both *Spook* and *Probable cytochrome p450 49a1* displayed conserved domains form the p450 superfamily, including the shared conserved domains pfam00067 and COG2124. *Apolipoprotein D-like* function of transport of small molecules was confirmed by the conserved domain lipocalin-like domain (pfam08212). The *Hormone receptor 4* was characterized by the ligand binding domain of hormone receptors (smart00430 and pfam00104), including the ligand binding domain of orphan nuclear receptor Ecdysone-induced receptor DHR4-like (cd06953) and a zinc finger conserved domain (cd00202, pfam00320, COG5641).

**Table 5:** List of the candidate genes and their associated conserved domains.

Gene name	Accession No	Seq. description	Seq. Length	E-value	Conserved domain accession no
*Neverland*	MW556747	Cholesterol 7-desaturase	2525	0.00E+00	cd03531, pfam00355, COG4638, PLN02281, COG2146, TIGR03228
*Spook*	MW556746	Cytochrome P450 307a1-like	1954	0.00E+00	pfam00067, PLN02394, COG2124
*Apolipoprotein D-like*	MW556748	Apolipoprotein D-like	844	2.2E-53	COG3040, PRK10477, pfam08212
*Hormone receptor 4*	MW556749	Hormone receptor 4 isoform X1	5767	0.0E0	cd06953, smart00430, pfam00104, smart00401, cd00202, pfam00320, COG5641
*Probable cytochrome p450 49a1*	MW556750	Probable cytochrome P450 49a1 isoform X2	3000	2.6E-112	pfam00067, COG2124, PLN02987, TIGR04538

**Fig 3:**
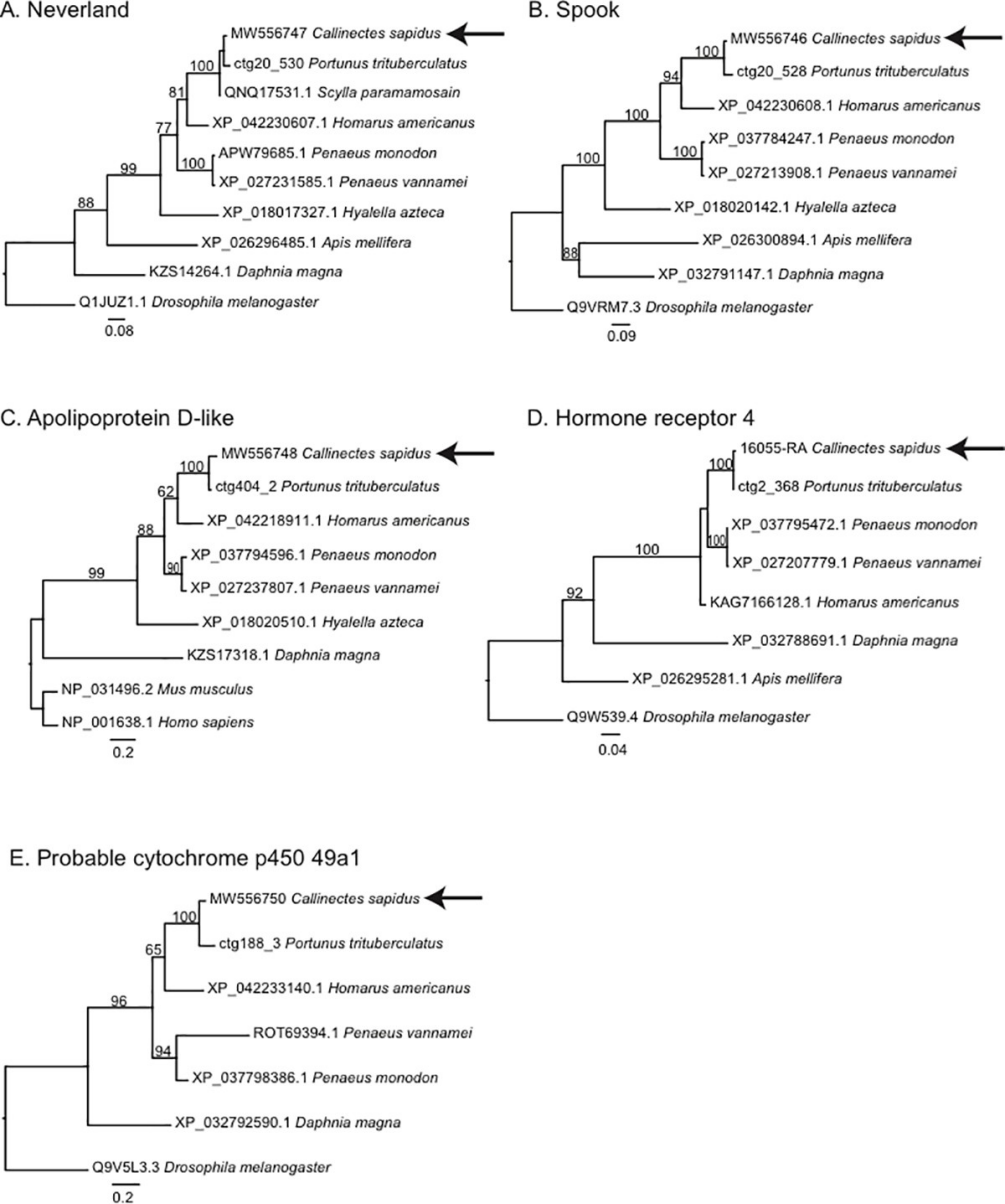
Phylogenic trees were generated using the default settings of Phylogeney.fr (http://www.phylogeny.fr/simple_phylogeny.cgi). Sequences were aligned using MUSCLE, trimmed with GBLOCKS, and trees were built with PhyML. The sequencing data is included in fasta format as supplemental files (S1–S5 Files). The *C*. *sapidus* sequences are noted with an arrow in A. *Neverland* (Accession No MW556747);B. *Spook* (Accession No MW556746);C. *Apolipoprotein D-like* (Accession No MW556748);D. *Hormone receptor 4* (Accession No MW556749);and E. *Probable cytochrome p450 49a1* (Accession No MW556750). Scale bars present fixed mutations per amino acid position. And, bootstrap values (%) supporting nodes are noted with numbers.

The putative amino acid sequence of *Neverland*, *Spook*, *Hormone receptor 4*, and *probable cytochrome p450 49a1* showed the highest similarity of the gazami crab, *P*. *trituberculatus* sequences (Fig 3A–3E). Such high identity was reflected in the phylogenetic trees where *C*. *sapidus* grouped with the other crab sequences in all five trees. The clade of *H*. *americanus* with the two crab species was found in four trees, albeit without strong support, followed by the two shrimp species. However, in the *Hormone Receptor 4* phylogeny, the *H*. *americanus* sequence was placed outside the crab shrimp clade, albeit without bootstrap support. *Callinectes sapidus*, the other decapods, and an amphipod species, *Hyalella Azteca*, were grouped closely in well-supported clades (96 to 100% support).

The putative amino acid sequence of *Neverland*, *Spook*, *Hormone receptor 4*, and *probable cytochrome p450 49a1* showed the highest similarity of the gazami crab, *P*. *trituberculatus* sequences (Fig 3A–3E). This high identity was reflected in the phylogenetic trees where *C*. *sapidus* grouped with the other crab sequences in all five trees. The clade of *H*. *americanus* with the two crab species was found in four of the trees, albeit without strong support, followed by the two shrimp species. However, in the *Hormone Receptor 4* phylogeny, the *H*. *americanus* sequence was placed outside the crab shrimp clade, albeit without bootstrap support. *Callinectes sapidus*, the other decapods and an amphipod species, *Hyalella azteca* were grouped closely in well-supported clades (96 to 100% support). Interestingly, *Daphnia magna* sequences were separated from crustaceans and were located close to insects (Fig 3A–3E). *Drosophila melanogaster* sequences were located as an outgroup in four of five genes (Fig 3A, 3B, 3D and 3E). For the Apolipoprotein D-like sequence, *D*. *melanogaster* and *Apis mellifera* sequences were not conserved in alignments, so the outgroup of *Mus musculus* and *Homo sapiens* were used.

Interestingly, *Daphnia magna* sequences were separated from crustaceans and were located close to insects (Fig 3A–3E). *Drosophila melanogaster* sequences were located as an outgroup in four of five genes (Fig 3A, 3B, 3D and 3E). For the Apolipoprotein D-like sequence, *D*. *melanogaster* and *Apis mellifera* sequences were not conserved in alignments, so the outgroup of *Mus musculus* and *Homo sapiens* were used.

### Candidate genes tissue distribution in the blue crab *C*. *sapidus*

**Fig 4:**
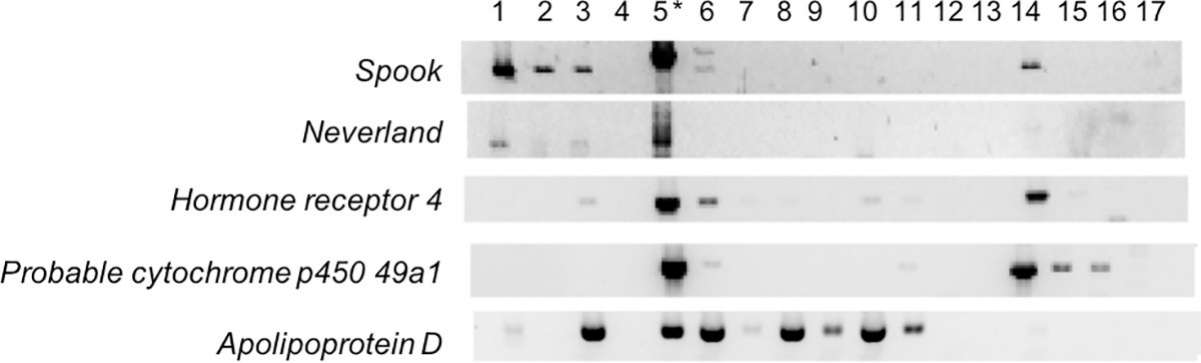
Spatial distribution of five candidate genes (*Spook*, *Neverland*, *Hormone receptor 4*, *probable cytochrome p450 49a1* and *Apolipoprotein D-like*) in the tissues of an adult female blue crab. Each cDNA tissue sample containing 12.5 ng total RNA equivalent was amplified by PCR. The PCR products were analyzed on 1.5% agarose gel and stained with ethidium bromide. The tissues are noted as: 1 = eyestalk, 2 = thoracic ganglia complex, 3 = brain, 4 = pericardial organ, 5* = Y-organ, 6 = mandibular organ, 7 = hepatopancreas, 8 = foregut, 9 = hindgut, 10 = antennal gland, 11 = gill, 12 = abdominal muscle, 13 = hypodermis, 14 = ovary, 15 = spermatheca, 16 = heart, 17 = hemocytes. Further expression analyses were carried out using the YO cDNAs (5*).

The expression of five candidate genes were confirmed using endpoint PCR across 17 different tissues (Fig 4) with *Spook*, *Neverland*, *Hormone receptor 4*, *probable cytochrome p450 49a1*, and *Apolipoprotein D-like* showing expression in the YO. Interestingly, the expression of *Spook* and *Neverland* was also noted in the EG, thoracic ganglia complex, and brain. *Hormone receptor 4*, *Spook*, and *probable cytochrome p450 49a1* were expressed in the ovaries. *Apolipoprotein D-like* had a wide distribution among the tissues tested.

### Changes in circulating ecdysteroid and cholesterol in the hemolymph of intact and ablated animals at different molt stages

**Fig 5:**
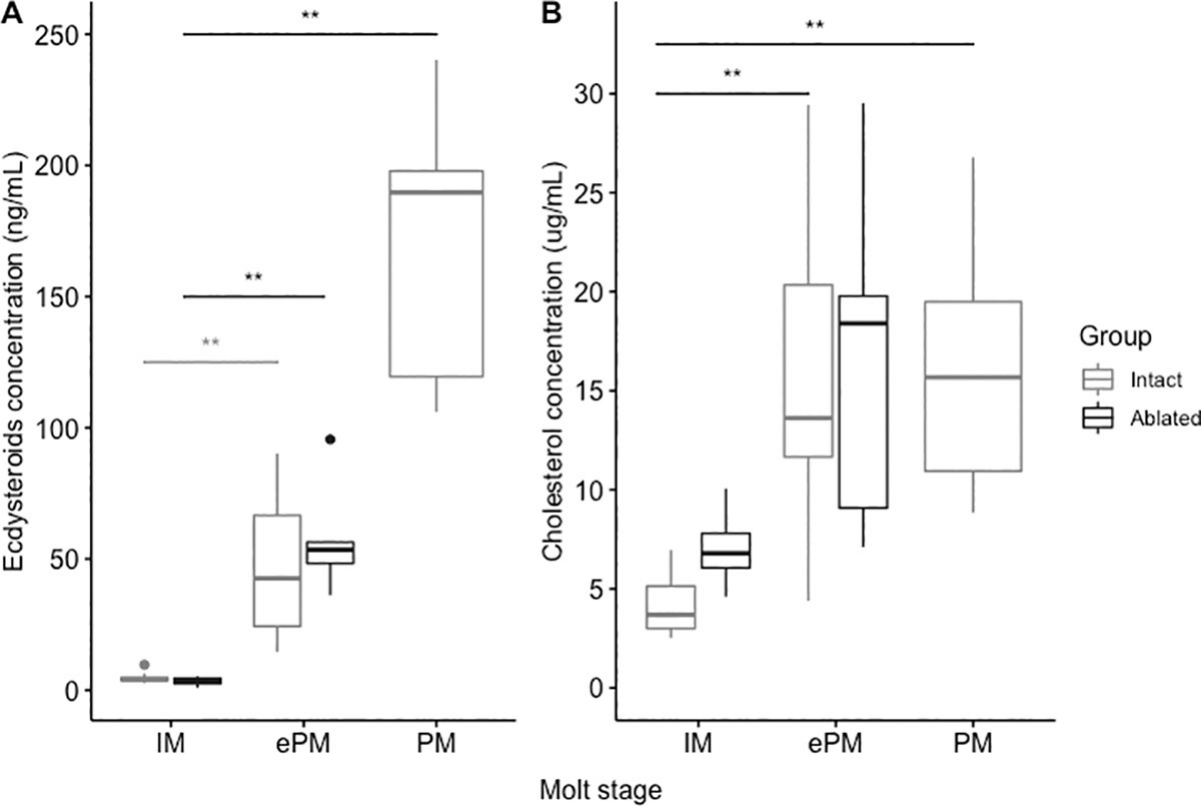
Levels of hemolymph Ecd and cholesterol at different molt stages. The group of animals undergoing natural molt cycle are colored in light grey (Intact) while the group of eyestalk-ablated animals are colored in dark grey (Ablated). (A) Ecdysteroid concentrations (ng/ml) in the hemolymph from intact prepubertal females at intermolt (IM), early premolt (ePM) and intermediate premolt (PM) and the corresponding stages from eyestalk-ablated adult females (D7 after ablation). (B) Cholesterol (free and cholesteryl esters) concentrations (μg/ml) from intact prepubertal females at intermolt (IM), early premolt (ePM) and premolt (PM) and the corresponding stages from eyestalk-ablated adult females (D7 after ablation). The levels of both Ecd and cholesterol were not statistically different at IM and ePM between natural and ablated animals (*P*>0.05, Students’ t-test). Statistical difference between IM, ePM and PM in a natural molt stage was evaluated using non-parametric Kruskall-wallis and Dunn test post-hoc (*P*<0.05). The dots represented outliers in the dataset.

The Ecd levels in the hemolymph of intact normal animals were determined at three stages: IM, ePM, and mid-PM. The hemolymph had significantly low levels of Ecd at IM with 4.8 ng/mL ± 2.1 ng/mL (n = 8); higher levels at ePM (47.1 ng/mL ± 29.0 ng/mL) (n = 7) and the highest level at 169.7 ng/mL ± 50. 5 ng/mL (n = 11) (Fig 5A).

The iePM (three days after eyestalk-ablation) showed significantly higher hemolymph Ecd (58.0 ng/mL ± 22.4 ng/mL, n = 5, *P*<0.05) than IM (3.4 ng/mL ± 1.8 ng/mL, n = 5), The elevated Ecd at iePM was similar to intact animals at ePM stage (47.1 ng/mL ± 29.0 ng/mL, n = 7; Fig 5A).

Interestingly, eyestalk-ablation caused a similar pattern for the cholesterol concentration in the hemolymph with 7.1 μg/mL ± 2.3 μg/mL at IM (n = 4) and 16.8 μg/mL ± 9.0 μg/mL at ePM (n = 5) (Fig 5B). The levels were similar to those of the intact animals (4.2 μg/mL ± 1.6 μg/mL at IM (n = 8) and 16.1 μg/mL ± 7.4 μg/mL at ePM (n = 15)). The cholesterol concentrations in the intact animals at PM remained similar to ePM (15.6 μg/mL ± 5.7 μg/mL) (n = 18).

### Candidate gene expression in the YO after eyestalk-ablation

**Fig 6:**
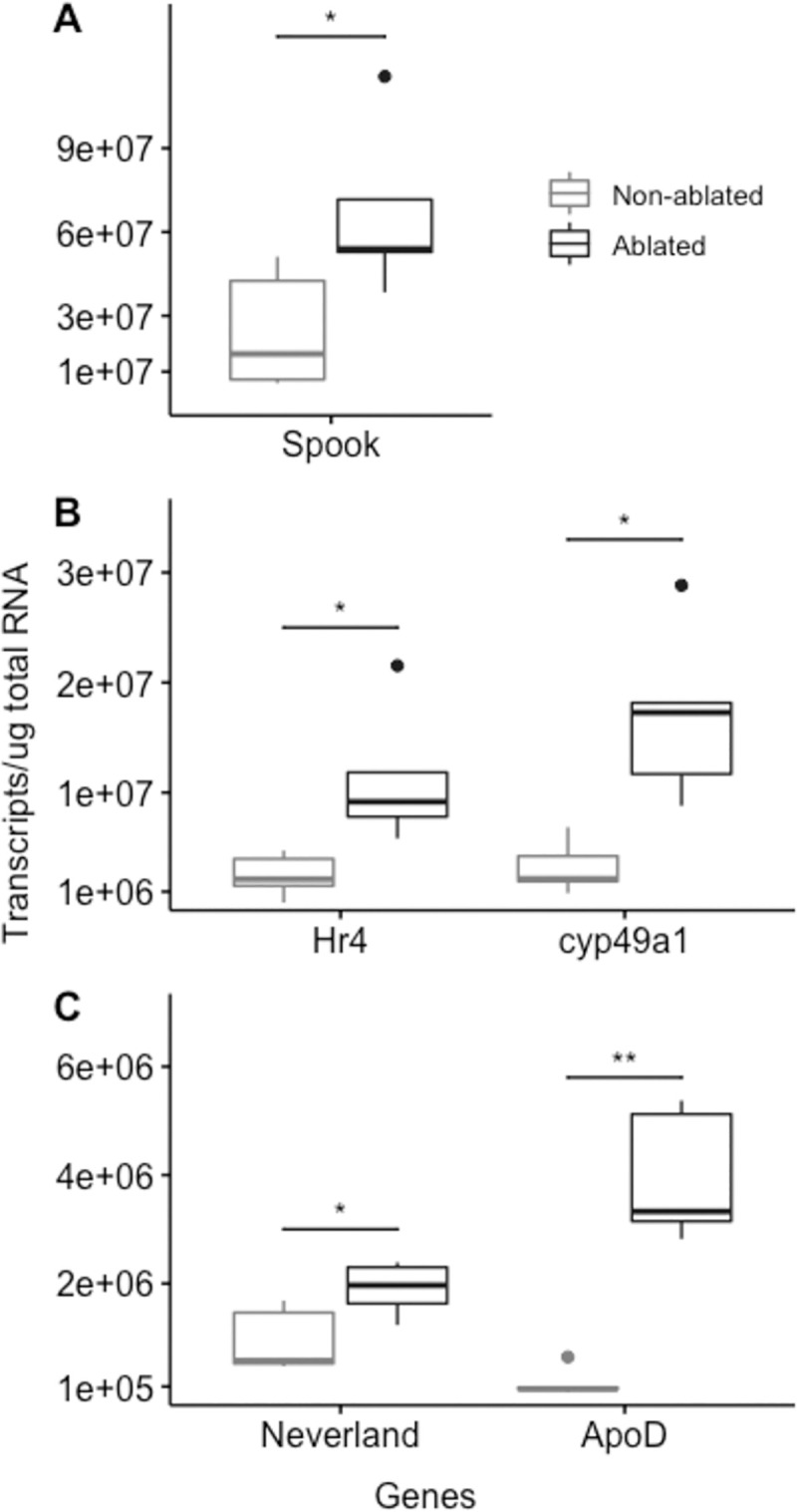
Boxplot representation of the candidate gene expressions in the YO of non-ablated animals (IM) and eyestalk ablated *C*. *sapidus* (iePM). Gene expression was measured by absolute qPCR assays and presented as the number of transcripts/μg total RNA. Non-ablated control animals are shown in light grey, eyestalk-ablated animals (ablated) in dark grey. (A) Expression of *Spook*. (B) Expression of *Hormone receptor 4 (Hr4)* and *probable cytochrome p450 49a1* (*cyp49a1)*. (C) Expression of *Neverland* and *Apolipoprotein D-like (ApoD)*. The expression levels of each gene measured in non-ablated and ablated samples were examined for statistical significance by Student’s *t*-test. Statistical differences were noted as”*” *P*<0.05, “**” *P*<0.01. The dots represented outliers in the dataset.

An eyestalk-ablation experiment was carried out to examine if the elevated Ecd was related to gene expression using a qPCR assay with the primers listed in Table 2. *Spook* presented the highest transcripts/μg total RNA in the YO (Fig 6A).

Elevated Ecd after eyestalk-ablation coincided with significantly increased expression levels of the five candidate genes; *Spook* (*P*<0.05), *Hormone receptor 4 (Hr4*; *P*<0.05*)*, *probable cytochrome p450 49a1 (cyp49a1*; *P*<0.05*)*, *Neverland* (*P*<0.05) and *Apolipoprotein D-lik*e (*ApoD*; *P*<0.01) in the YO, compared to crabs at IM (non-ablated individuals). *Apolipoprotein D-like* showed the most prominent change in copy number per μg total RNA (n = 5) with 1.7e05 ± 2.7e05 at IM to 4.0e06 ± 1.2e06 at iePM, followed by *cyp49a1*: 3.2 e06 ± 2.4e06 at IM to 1.7e07 ± 0.8e07 at iePM; *Hr4*: 2.5 e06 ± 1.9e06 at IM to 1.1 e07 ± 0.6e07 at iePM; *Spook*: 2.5e07 ± 2.1e07 at IM to 6.7e07 ± 3.0e07 at iePM; and *Neverland*: 9.4e05 ± 5.8e05 at IM to 1.9e06 ± 0.5e06 at iePM (Fig 6).

### Candidate gene expression in intact animals at different molt stages

**Fig 7:**
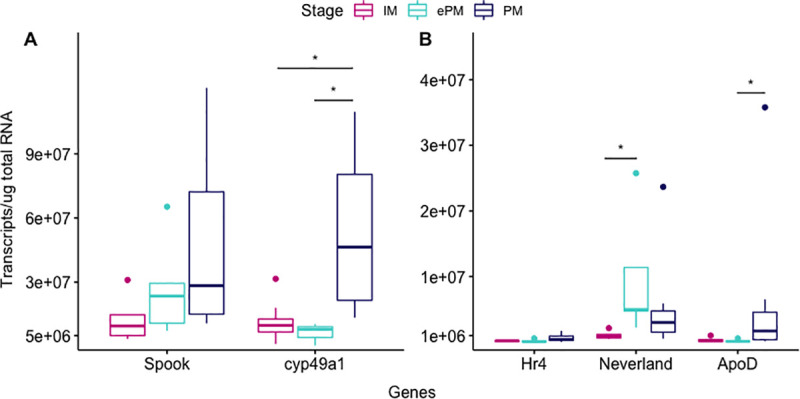
Boxplot representation of the candidate gene expressions in the YO of intact *C*. *sapidus* at different molt stages. Gene expression was measured by absolute qPCR assays and expressed in the number of transcripts/μg total RNA. Molt stages are colored as followed: IM (dark pink), ePM (turquoise), and PM (dark blue). (A) Expression of *Spook* and *probable cytochrome p450 49a1(cyp49a1)*. (B) Expression of *Hormone receptor 4 (Hr4)*, *Neverland*, and *Apoprotein D-like (ApoD)*. The expression levels measured at the three molt stages were analyzed using a non-parametric one-way ANOVA Kruskal-Wallis followed by a Dunn post hoc test. Statistical differences were noted as”*” *P*<0.05. The dots represented outliers in the dataset.

The validation of upregulated gene expression at iePM was carried out with animals undergoing a natural molt cycle at IM (n = 8), ePM (n = 6) and PM (n = 8). In this experiment, two genes, *Spook* and *probable cytochrome p450 49a1 (cyp49a1)*, had the highest expression in the YO at PM (Fig 7A).

The increase observed from IM to ePM to PM for *Spook* was not statistically significant (IM: 1.3e07 ± 1.1e07, ePM: 2.7e07 ± 2.3e07, PM: 4.8e07 ± 4.4e07 transcripts/μg total RNA). There was no statistical change between IM and ePM for *cyp49a1*; however, its expression increased significantly at PM (IM: 1.1e07 ± 1.0e07, ePM: 6.2e06 ± 4.7e06, PM: 5.3e07 ± 4.2e07 transcripts/μg total RNA; *P*<0.05). *Hormone receptor 4*, *Neverland* and *Apolipoprotein D-like* showed the lowest expression, regarding of the molt stage (Fig 7B). Statistically significant increases were observed for *Neverland* between IM and ePM (1.1e06 ± 0.7e06 to 9.8e06 ± 9.4e06 transcripts/μg total RNA; *P*<0.05) and *Apolipoprotein D-lik*e between ePM and PM (1.8e05 ± 2.0e05 to 6.8e06 ± 1.2e07 transcripts/μg total RNA; *P*<0.05).

### Summary of the ecdysteroidogenesis pathway

**Fig 8:**
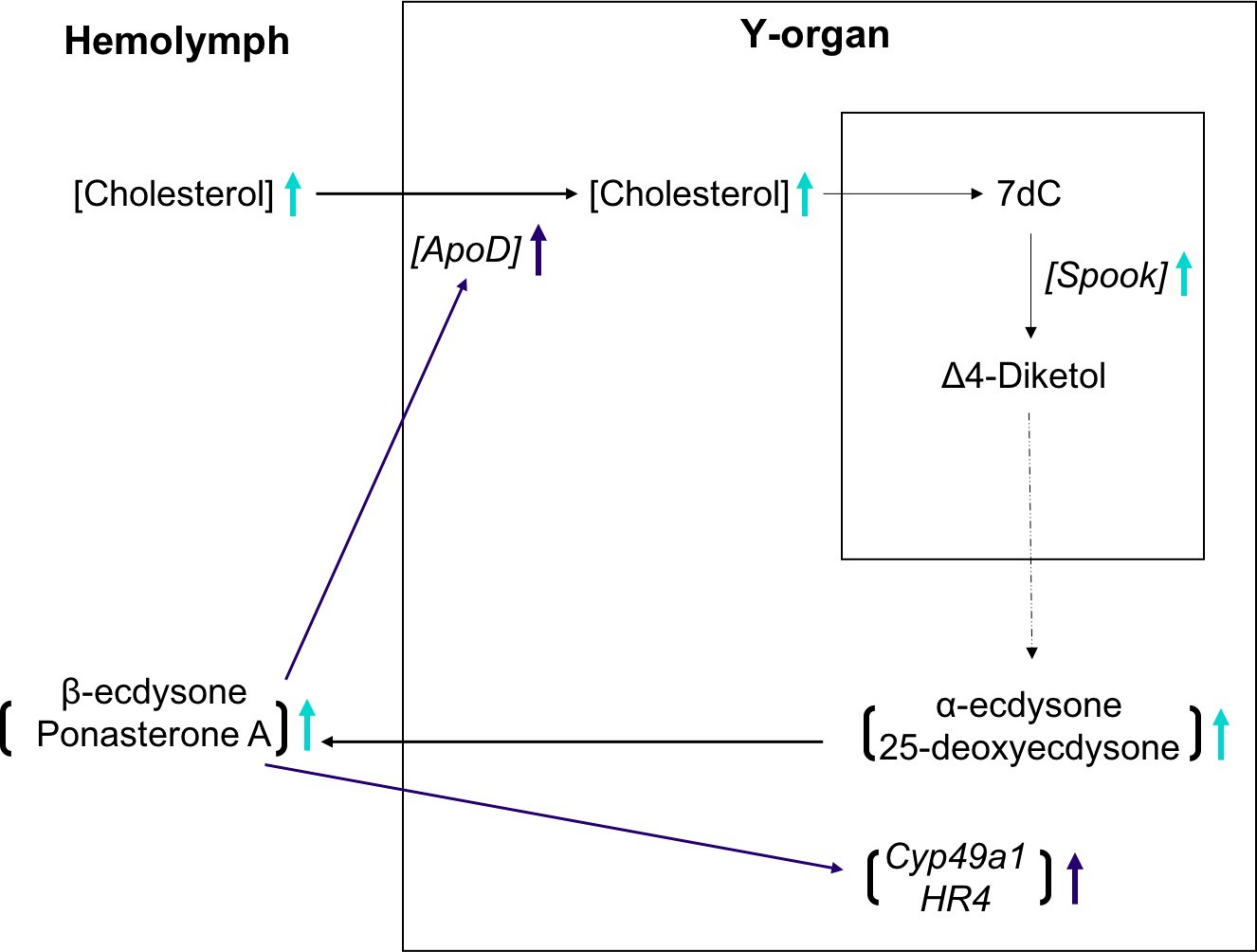
Summary of the ecdysteroidogenesis pathway in *C*. *sapidus* based on the data presented in this study. First, cholesterol concentration increased at early premolt (ePM, turquoise in Fig 7), resulting in the initial rise in ecdysone synthesis and release via upregulation of *Neverland* (*Nvd)* and *Spook*. Then this initially elevated ecdysone in hemolymph upregulated *Apolipoprotein D-like* (*ApoD*) at premolt (PM, dark blue, in Fig 7) for further increase in cholesterol transport for ecdysteroid synthesis. It also upregulated other ecdysone responsive genes, including *probable cytochrome p450 49a1* (*cyp49a1*) and *Hormone receptor 4* (*HR4*) at PM. The dashed line represents the rest of the ecdysteroidogenesis steps not included in this study. The upward arrows illustrate the upregulation. 7dC: 7-dehydrocholesterol.

Based on the results from this study, we proposed the following summary of the ecdysteroidogenesis pathway in *C*. *sapidus*. First, the cholesterol concentration increased at ePM (turquoise arrow), resulting in the initial rise in ecdysone synthesis and release, via the upregulation of *Neverland* (*Nvd)* and *Spook* (Fig 8) at ePM. Then this elevated ecdysone concentration in hemolymph upregulated *Apolipoprotein D-like* (*ApoD*) at PM (dark blue arrow) to further accelerate the transport of cholesterol for ecdysteroid synthesis. The increased ecdysteroid concentration also up-regulated other ecdysone responsive genes, including *probable cytochrome p450 49a1* (*cyp49a1*) and *Hormone receptor 4* (*HR4*) at PM.

## Discussion

The present study describes the identification and changes in the expression of candidate genes involved in ecdysteroidogenesis and cholesterol uptake in YO of the blue crab, *C*. *sapidus*. The candidate genes were identified using RNAseq analysis of the YO transcriptomes at two different molt stages: IM and iePM. Their expression in the YO is discussed in the context of the increase of hemolymph Ecd and cholesterol at iePM and ePM.

The transcriptomic analysis allows the functional genomics study of the critical physiological processes of non-model organisms, including decapod crustaceans. Specifically, transcriptomic analysis has been adopted to unravel the molecular mechanisms governing molting in the whiteleg shrimp, *Litopenaeus vannamei* [46], the Chinese mitten crab, *Eriocheir sinensis* [47], the land crab, *Gecarcinus lateralis* [48–50] and the Danube crayfish, *Pontastacus leptodactylus* [51]. A first YO transcriptome of intact animals has been sequenced in the blue crab *C*. *sapidus* [52] but further investigation is required to better understand the mechanism of the molting process, specifically by which the levels of Ecd are regulated during the molt cycle.

In the current study, the sequencing data for gene discovery were generated using the libraries obtained from pooled YO RNA samples from multiple individuals per molt stage. It is not unusual to use pooled RNA samples for sequencing. A similar approach has been used for gene discovery and identifying specific candidate genes in *C*. *sapidus* [53], the greentail prawn, *Metapenaeus bennettae* [54], or the giant river prawn, *Macrobrachium rosenbergii* [55]. A non-replicated study can be used as a pilot study to test a proposed hypothesis and be validated by targeted gene-expression or further replicated study [56].

The high quality of YO transcriptome is supported with ~96% of completed BUSCOs for the clustered assembly that validates the completeness and accuracy of our transcriptomic data. The reduction of duplicated BUSCOs and the increase of single-copy BUSCOs confirm the use of CD-HIT-EST to reduce redundancy. These results agree with other BUSCO analyses carried out in crustaceans such as the greentail prawn, *M*. *bennettae* [54], and the black tiger shrimp, *Penaeus monodon* [57]. Moreover, the Trinity assembly presents a high N50 (2,854), which implies the presence of isoforms. The clustered Trinity_95 showing a reduced N50 (1,510) after CD-HIT-EST confirms the redundancy improvement, which is similar to the N50 (1537.79) of the YO filtered transcriptome of *G*. *lateralis* [48].

As expected, when using a TPM threshold of 1 more contigs are found in the YO at iePM than IM, corroborating the elevated YO transcriptional activity. Overall, the number of contigs (40,605 at IM and 46,240 at iePM, respectively) obtained in this study is similar to the filtered YO transcriptome in *G*. *lateralis* (48,590) [48] and to the 31,661 predicted protein coding region in the intact *C*. *sapidus* [52]. As a first exploration, our work was focused on highly abundant transcripts (TPM) in the YO at iePM. All the sequences of interest belonged to the 33,609 transcripts shared by the two transcriptomes and presented a higher abundance (TPM) at iePM than IM.

The 608 most abundant sequences (TPM>100) were screened to identify candidate genes. Through functional annotation and based on insect research, the genes possibly regulating ecdysteroid levels in YO were identified, specifically those that are involved in the ecdysteroidogenesis (*Neverland*, *spook*), ecdysone responsive genes (*cyp49a1*, *Hr4*), and involved in the cholesterol transport and uptake (*ApoD*).

The involvement of Halloween genes (cytochrome p450 family) and a Rieske domain oxygenase in ecdysteroidogenesis have been well-described in insects [20,21,58–61]. Orthologous genes identified in *Daphnia pulex* [25] underscore the functional importance and conservation of this pathway in the arthropod phylum. Homology for these five genes is determined based on phylogenetic analysis, which shows in most cases a clear decapod and crustacean group. These sequencing trees are generally consistent with taxonomic relationships in most cases. The first step has been described similarly in insect and crustaceans. First, the 7,8-dehydrogenase, *Neverland*, converts cholesterol into 7dC. The presence of *Neverland* has been identified in the YO of the black tiger shrimp, *P*. *monodon* [62], and the land crab, *G*. *lateralis* [49]. The next step, from 7dC to Δ4-Diketol, remains to be unraveled in crustaceans and is referred as the ‘Black box’, but the Halloween gene *Spook* is involved in this step in insects [23].

The molt cycle is regulated by an interplay between ecdysteroid and neuropeptide hormones (such as MIH and CHH [8]). YO is the tissue for ecdysteroidogenesis in decapod crustaceans and displays the exclusive MIH binding site [63]. However, *Neverland* and *Spook* expression in *C*. *sapidus* is not exclusive to the YO. Expression of these genes was observed in other tissues such as the EG, thoracic ganglia complex, brain, and ovary. A similar pattern has been observed in *P*. *trituberculatus* [26], but further study of the localization and translation of mRNAs in these tissues is needed. *Neverland* and *Spook* have been identified in ovarian cells in other species such as the salmon louse, *Lepoeophtheirus salmonis* [64], the water flea, *D*. *magna* [65], the acari, *Ornithodoros moubata* [66], the mosquito, *Anopheles gambiae* [67], and the fruit fly, *Drosophila melanogaster* [68]. In *D*. *magna*, two tissue-specific paralogs of *Neverland* are found in the gut epithelium (*Neverland*1) and in the ovary (*Neverland*2) [65]. The expression of *Neverland* and *Spook* in ovarian tissue in *C*. *sapidus* could be maternal RNAs for Ecd synthesis translated during embryogenesis, as an earlier report states that crustacean ovaries contain maternal Ecd [69]. Alternatively, the ovary may be another site for ecdysteroidogenesis in *C*. *sapidus* for ovarian development that takes place multiple times during adulthood. Adult females experience puberty-terminal molt after which the high levels of MIH in the hemolymph inhibits the YO activity to maintain low hemolymph Ecd at the permanent IM [70].

Expression of the candidate genes possibly responsible for the changes in Ecd levels was further confirmed using qPCR assays. A significant upregulation of *Neverland* expression in the YO at iePM after eyestalk-ablation is also seen in the intact animals at the same molt stage. Interestingly, *Neverland* presents the highest expression at ePM followed by a decrease at PM. A similar expression pattern is observed during the molt cycle in the YO of the black tiger shrimp, *P*. *monodon* [62]. Since the conversion of cholesterol to 7dC is required for further Ecd production at PM, it is proposed that the translated *Neverland* may have a long half-life. The presence of MIH keeps the Ecd low during IM by suppressing YO activity. Hence, it is plausible to suggest that the elevated MIH levels at ePM may negatively affect *Neverland* expression [8]. The increase of cholesterol concentration observed in the hemolymph at ePM in *C*. *sapidus* supports its recruitment for ecdysteroidogenesis. Like *Neverland*, upregulation of *Spook* expression is seen in the YO at iePM. This induction after eyestalk-ablation was not observed in the YO of *P*. *trituberculatus* [26]. Despite the lack of statistical significance, *Spook* presents a higher expression in *C*. *sapidus* at ePM and PM in a natural molt cycle. A similar increase in ePM has been demonstrated in YO of *G*. *lateralis* [49]. The increased Ecd in the hemolymph at ePM (or iePM) and PM, confirms the production of the molting hormone by the YO. Interestingly, *Spook* exhibits the highest expression in both qPCR assays and transcriptome analysis in *C*. *sapidus*. This result implies that *Spook* or the ’Black box’ step (from 7dC to Δ4-Diketol) may be a key step in ecdysteroidogenesis.

The function of cytochrome P450 includes not only steroid synthesis and breakdown but also clearance of fatty acids and xenobiotics. While the function of *cyp49a1* remains to be elucidated in *C*. *sapidus*, *cyp49a1* shows high expression in both YO and ovary together along with a slight expression in the spermatheca and heart. Similar to the expression of *Neverland* and *Spook*, the up-regulation of *cyp49a1* expression is observed at iePM after eyestalk-ablation and at PM during a natural molt cycle. In *A*. *gambiae*, *cyp49a1* expression was higher in the pupae stage rather than the adult mosquito [71] suggesting a role of *cyp49a1* in arthropod development at a life-stage dependent manner. It is important to note in the present study that the tissues associated with the adult stage, i.e., ovarian and spermathecal tissues, were developing in the animals undergoing pubertal terminal molt. This suggests that *cyp49a1* could be involved in the development of *C*. *sapidus*.

Ecdysteroid regulates arthropod (insects and crustaceans) growth by signaling through a heterodimer receptor of EcR and RXR/Ultraspiracle (USP). The binding of ecdysone to the EcR-RXR/USP triggers a cascade of Ecd-responsive genes including *Hormone receptor 4* [59]. The involvement of *Hormone receptor 4* seems to be limited to metamorphosis in *Drosophila* [72,73] and ecdysis in the German cockroach, *Blatella germanica* [74]. In the Chinese mitten crab, *Eriocheir sinensis*, *Hormone receptor 4* expression differs between the zoeal and megalopal stages, highlighting its role in crustacean metamorphosis [75]. In *C*. *sapidus*, *Hormone receptor 4* was mostly expressed in the YO and ovary with a low detection in the mandibular organ and brain. Its expression was induced in the YO after eyestalk-ablation. The change in expression, though non-significant during the prepurberty molt cycle, shows a slight increase at PM. In order to have a better understanding of the role played by *Hormone receptor 4* in *C*. *sapidus*, it would be interesting to investigate expression immediately after eyestalk ablation.

Unlike vertebrates, arthropods do not *de novo* synthesize the cholesterol needed to produce Ecd. Transport and cholesterol uptake may be limiting steps for ecdysteroidogenesis. Cholesterol is transported in the hemolymph into lipoproteins [18,76], which are in turn associated with apolipoproteins and involved in the lipoprotein membrane integrity and in binding to receptors [76]. We identified *Apolipoprotein D-like* expression in different tissues in the blue crab including the YO. An increase in *Apolipoprotein D-like* expression by qPCR after eyestalk-ablation was confirmed by an up-regulation at PM in a natural molt cycle, implicating *Apolipoprotein D-like* in cholesterol uptake by the YO in *C*. *sapidus*.

Increased cholesterol concentrations in the hemolymph of *C*. *sapidus* at iePM and at ePM in a normal molt cycle provide the YO with the precursor needed to increase the production of Ecd. While Ecd production by the YO reaches its maximum at PM [4], the cholesterol levels remain similar in ePM and PM. This suggests that there may be some differences in the rate of cholesterol transport in the hemolymph and uptake by the YO. The YO at PM may increase cholesterol uptake while maintaining the necessary amounts of hemolymph cholesterol at ePM levels. This notion is supported by studies that demonstrated radiolabeled cholesterol accumulated the most in the YO while a small increase was observed in the hemolymph suggesting an active uptake by the YO [14,15].

## Conclusions

This study presents an exploration of the blue crab *C*. *sapidus* YO transcriptome during normal and induced molt cycles to identify candidate genes in the ecdysteroid synthesis, cholesterol uptake and transport, and the characterization of their expression by qPCR. Ecdysteroidogenesis genes (*Neverland*) and members of the Halloween genes (*Spook*, *Disembodied*, and *Shadow*) have been identified in the YO transcriptomes, and the expression of the early ecdysteroid synthesis steps (*Neverland* and *Spook*) was characterized during a natural molt cycle. The expression of two ecdysone-responsive genes (*probable cytochrome p450 49a1* and *Hormone receptor 4*) increased accordingly with the Ecd production. A similar pattern was observed with *Apolipoprotein D-like* and the cholesterol concentration in the hemolymph, suggesting its role in the uptake of the latter by YO. Overall, key genes with increased transcripts at ePM, along with elevated cholesterol in the hemolymph, corroborate the elevated Ecd synthesis by the YO as summarized in Fig 8, which overall supports the earlier finding of the presence of positive feedback of Ecd on YO [8]. The transcriptome and expression data are described in this paper. The function of each gene at the translation and protein level remains to be studied in the future.

## Supporting information

S1 Table Quantitative RT-PCR standard curve parameters. (XLSX)

S1 File *Neverland* sequences used for alignment and phylogeny. (FASTA)

S2 File *Spook* sequences used for alignment and phylogeny. (FASTA)

S3 File *Apolipoprotein D-like* sequences used for alignment and phylogeny. (FASTA)

S4 File *Hormone receptor 4* sequences used for alignment and phylogeny. (FASTA)

S5 File *Probable cytochrome p45049a1* sequences used for alignment and phylogeny. (FASTA)
